# Exploring the motivational roots of farmers’ adaptation to climate change‑induced water stress through incentives or norms

**DOI:** 10.1038/s41598-022-19384-1

**Published:** 2022-09-08

**Authors:** Tahereh Zobeidi, Jafar Yaghoubi, Masoud Yazdanpanah

**Affiliations:** 1 Department of Agricultural Extension, Communication and Rural Development, University of Zanjan, Zanjan, Iran; 2 Department of Agricultural Extension and Education, Agricultural Sciences and Natural Resources University of Khuzestan, Khuzestan, Iran

**Keywords:** Environmental social sciences, Psychology, Human behaviour

## Abstract

The aim of the current study is to consider farmers' perceptions regarding the impacts of climate change on water resources and their intention toward adaptation in southwestern Iran. To this end, this study applied the theory of reasoned action and the norm activation model as well as these two models in combination. A descriptive quantitative research study was designed and conducted using cross-sectional survey methods among 250 farmers in Khuzestan province in southwestern Iran, selected through multistage sampling methods. Research data were collected through a structured questionnaire whose validity was confirmed by a panel of experts; scale reliability of the questionnaire was approved through a pilot study. Structural equation modeling analysis revealed that the norm activation model, the theory of reasoned action, and a model integrating the two can predict 32, 42, and 47%, respectively, of changes in farmers' intention toward performing climate-change adaptation activities. In the combined model, personal norm, subjective norm, and attitude were able to influence the farmers’ intention to perform adaptive behaviors. Attitude towards adaptation is the most powerful predictor in explaining intention to adaptation. Subjective norm is the most important predictors of moral norms which is the logical confirmation behind the combination of the two models. In addition, the combined model has better predicting powerful that each model separately. The research findings hold valuable implications for policymakers seeking to increase the intention of farmers to implement adaptation activities against a background of harsh climate change and water scarcity in this region of Iran.

## Introduction

There is strong scientific proof, backed by a plethora of studies, to support the existence of climate change. A grave challenge with negative effects on humans and the natural environment^1^, this phenomenon is already posing numerous challenges to economic, social, and environmental sectors globally^2^. The effects of climate change on the agricultural sector are unique, and the leading cause of economic and physical suffering in developing countries^2,3^ which have weaker economies and inadequate economic, physical, and social infrastructure^4^. The most significant aspect of this crisis has been the reduction in farm production, and the most important factor in this regard—along with other variables such as decreased soil quality and increased disease and pests—is the changes caused by climate change in the quantity and quality of water resources^5^. Climate variability is expected to disrupt the reliability and sustainability of the agricultural sector^6^, with changes expected to intensify in the future. By 2050 an estimated two-thirds of the world's population could face water stress due to climate change^7^. Climate change affects the hydrological cycle in a number of ways that may overshadow long-term and short-term access to water resources in many areas^6,8^. Climate variability affects the quantity, quality, and spatio-temporal distribution of existing water resources, and has probable negative impacts in three categories: water supply, flooding, and pollution^9^. Climate change disrupts hydrological systems by influencing the amount and pattern of rainfall and the melting of snow and ice, thus affecting water resources both quantitatively and qualitatively. Climate change is also having a negative impact on runoff and surface water and on groundwater resources due to rising temperatures and their impact on glaciers^10^. In agriculture, rising temperatures can affect cropping seasons, increase evapotranspiration, alter irrigation requirements, and cause heat stress^11^. In this regard Schewe et al^12^ used statistical models to estimate that for every one-degree increase in temperature, 7% of the world's population lose 20% of their renewable water resources. Clearly, therefore, agriculture, the largest consumer of water in the world, is affected by climate change, through changes both in water access and water demand^13^, and this can directly reduce farmers' production and income, especially in arid and semi-arid regions^6^.

This situation has made climate change and its effects on water resources and agricultural products a "hot topic" of global concern. In the face of adverse climate change impact problems, adaptation is required at all levels, and there is an increasing call for the management of agricultural water as part of adaptation measures^14^.

Farmers, due to their decision-making role in land use and resource allocation on farms have a critical influence regarding adaptation to the threats and opportunities posed by a changing climate^14,15^. In fact, without farmers’ willingness to adapt, government policy regarding adaptation measures will remain "on paper" and will never be implemented at the farm level^1^. There is strong evidence, however, that informed, successful, and fully implemented adaptation strategies significantly alter the negative effects of climate change on agriculture^16^. In fact, no sector of the economy has more at stake with respect to successful adaptation responses than agriculture^17^. Therefore, governments and policymakers need to pay special attention to managing and planning for climate change with a view to exploring the adaptive capacity of farmers and supporting them in this situation. The first step for governments is to recognize the reality of the current situation and examine how to take farmers’ current motivation, priorities, and adaptation behavior into account in the formulation of appropriate adaptation policies^17,18^. This has led to a great deal of emphasis being placed on understanding the adaptive behavior of farmers at the local level^19^. However, there is a growing body of empirical evidence that has addressed to identify psychosocial determinants behind water conservation and adaptive behaviors^20,21^, prior research in Iran are poorly known on adaptation behavior in the agriculture sector^22^. Therefore, there is a vast research gap and more empirical studies are thus needed to adequately comprehend psychosocial determinants underlying the intention to adopt adaptive behavior, especially in Iran. Hence, the main aim of the current study was to bridge the existing research gap by comprehensively determining and elaborating intentions to adopt adaptive behavior by farmers. In particular, the aim of the current study is thus to consider farmers' perceptions regarding the impacts of climate change on water resources and their intention toward adaptation in Khuzestan province in southwestern Iran. This province is among the areas most vulnerable to climate change impacts. Our study focused on farmers’ willingness to adapt their behavior. There is a large volume of past research on linking norms and individual intention^23^, and intentions are a good proxy for actual behavior^24^. We employ social-psychological models to understand and predict farmers' intention and environmental behavior^25,26^. The models provide useful operationalizations of the theoretical variables utilized and specify the causal courses through which they affect behavior^25^. This study, of the various theories recommended in this regard (see Mitter et al^26^; Chen^27^) will apply two models: the theory of reasoned action (TRA; e.g., Ajzen^28^) and the norm activation model (NAM; Schwartz^29^) as well as these two models in combination. We apply these two models because of their proven success in predicting adaptation and mitigation behaviors globally (Chen^30^; Masud et al^31^). Norms are important drivers of pro-environmental behavior^23^ such as climate change adaptation, and both models include norms. Although the main goal of the current study is to determine the factors affecting farmers' willingness to carry out adaptation activities, the research also has a secondary goal including to discover, whether the decision to perform climate adaptation behaviors is based on normative, moral behaviors (under the norm activation model) or led mainly by the calculation of personal utility and costs (theory of reasoned action). In response to Bamberg et al^32^, the second goal was to compare these models (TRA and NAM) in predicting intention to environmental behavior. The paper's third goal is to attempt to integrate these models into one model. Combining the two theories is important because they each emphasize two different aspects of the values and inner desires of individuals^33^. Therefore, the current study is to consider farmers’ willingness to perform adaptation behavior by combining two models the NAM and the TRA.

### Theoretical framework

Schwartz^34^ designed and developed the norm activation model (NAM) to examine prosocial behavior (Han, 2015). Prosocial behavior refers to a person's actions based on their intention to help others and includes a wide range of helping, sharing, and cooperating behaviors^35,36^. This model has been used to shed light on activities planned to improve environmental problems^25^. NAM focuses on the factors that lead to altruistic behavior, a behavior that is based on individuals giving up their personal interests for the sake of the environment and society^29^.

NAM has three main elements, including: awareness of consequences (AC), ascription of responsibility (AR), and personal norms (PN), and these can be used to calculate whether prosocial or environmental behaviors are altruistic behaviors^34^. PN is the central construct that stimulated by other constructs including AC, AR, and even subjective norms (SN)^37^. The aim is for farmers to diagnose that climate variability is a problem/hazard for their property (water, land, crops) and needs tackling (PN), and that their actions will facilitate the issue (AC). PN is shaped by internalizing subjective norms by adapting them to the personal value system^29^. Therefore, subjective norms should impact PN as well (but in this study, only in the combined model).

The main premise of the NAM is that PN directly determines prosocial behaviors^38^. According to Schwartz^29^, PN represent the strong sense of moral duty that people experience that leads them to participate in prosocial behaviors. Accordingly, AC and AR determine the PN, and the PN, in turn, directly predicts behavior. AC refers to knowledge about the adverse environmental consequences of performing/not performing a particular behavior^39^. The AC of doing or not doing a behavior triggers the norm, as when people are informed about the negative consequences of their behavior for others, they are more likely to feel morally obligated^35^. AR refers to the sense of feeling responsibility for the negative consequences of not performing a prosocial act, and PN expresses the moral duty to perform or to refrain from performing certain actions^35,40^.

Many researchers in various fields have emphasized the importance of emotional processes (and have predicted positive and negative emotions) in their explanation of pro-environmental behavior and prosocial behavior as decisions involve emotional processes^40–43^. In these studies, the predicted feelings of pride and guilt, respectively, were used as an example of a positive and a negative emotion. Pride is something positive experienced, as a pleasant feeling and often bringing with it a sense of worth. Guilt, on the other hand, is a negative emotion, causing feelings of tension, regret, and worry^38,42^. The feelings of pride and guilt are referred to as conscious emotions, as they are evoked after following (or not following) personal or social norms. These personal and social criteria are often based on moral behavior; consequently, the self-conscious emotions evoked by these criteria trigger altruistic behavior. Integrating these basic concepts into the main framework of norm activation can provide a clearer understanding of the decision-making processes in the performance of environmental behaviors by individuals^40^. Empirical evidence shows that emotions such as pride and guilt can influence personal norms in the norm activation model^40,41^.

Some believe that performing pro-environmental behaviors is a moral and normative issue. Others believe that these behaviors can mainly be led by the calculation of personal utility and costs. This can explain by rational choice models. Accordingly, in this study, we apply one of the best known models of rational choice models, the theory of reasoned action (TRA).

**Figure 1:**
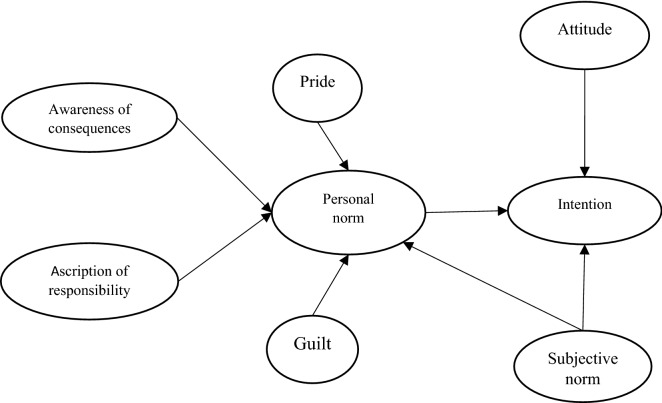
Integrative model of NAM and TRA.

TRA, set out by Ajzen^44^ is a general theory for predicting all types of deliberate social behavior and emphasizes the importance of cost–benefit reasoning when deciding to perform a social behavior; this in contrast with NAM which is limited to predicting moral behaviors^25^. In other words, TRA that later extended to theory of planned behavior (TPB) considers the individual in general as a utility-maximizing actor^28^. TRA consists of two main independent latent constructs (variables) including attitude and subjective norm, on which an intermediary variable, intention, is predicated. Intention, in turn, can directly predict behavior. Attitude refers to the fact that an individual, after comparing the benefits, costs, and risks associated with a target behavior, evaluates the extent to which the target behavior is positive or negative. In fact, an attitude refers to a person's favorable or unfavorable assessment of the target behavior^28,45^. Subjective norm is a social factor that is related to perceived social pressure to do or not to do a target behavior^46^. In summary, the framework of this research includes eight latent constructs and seven hypotheses (Fig. 1). The variables of awareness of results, attribution of responsibility, pride, guilt, and social norm are the determinants of moral norms. In turn, moral norms, along with attitudes and subjective norms, determine the intention to perform an adaptive behavior.

## Research method

### Participants

To achieve this goal, a descriptive quantitative research study using a cross-sectional survey was designed and conducted. The population of interest in this study was farmers in Khuzestan province in southwestern Iran. Khuzestan province is the most important producer of agricultural products in Iran. As well as being the largest wheat producer in Iran, Khuzestan has a significant share in the production of rice, vegetables including leafy vegetables, onions, garlic, squash, eggplant, okra, cabbage, and fruits including citrus, dates, and pomegranates.

### Instruments and data collection

The data were gathered using a structured questionnaire through face-to-face interview. To prepare the questionnaire, we conducted an extensive literature review on adaptation to climate change and the NAM and TRA theories, especially in the context of Iran. Previous studies were thus used as much as possible to select the items that measured the variables of the research model.

The research questionnaire had two main parts; the first consisted of items to measure the different variables under the NAM and TRA that were designed as a five-point Likert scale. The second part was related to individual and socioeconomic characteristics of farmers, and was open-ended. The interview with each farmer took about 30 min and was conducted at the farmer's home or on the farm. Before the interview, its purpose and related research were explained to farmers. Farmers were also assured that the data were confidential and anonymous. Response rates were above 90%, and farmers who turned down the interview for various reasons were replaced by new ones. Interviewees were selected from local people to facilitate communication with farmers and were provided with the necessary background training. Table 1 presents the survey items included in the questionnaire. The validity of the questionnaire was confirmed by a panel of experts. To investigate the scale's reliability, the questionnaire was pretested with 30 farmers in a pilot study. Cronbach's alpha coefficient was used to test the reliability of the constructs. As shown in Table 1, the minimum Cronbach's alpha coefficient was 0.741, which is higher than the recommended threshold of 0.7^47^.

**Table 1:** Scales, reliability and validity indices of latent constructs and sources.

Awareness of consequences (α = 0.681, CR = 0.914, AVE = 0.883)	Factor loading	Sources
Adaptation measures can prevent serious threats to the economy and my agricultural income	0.68	^36^
Adaptation measures can prevent losing income due to water scarcity	0.85	
An affective Adaptation measure will improve the health and well-being of me and my family	0.85	
Adaptation measures can prevent conflict among farmers induced by water scarcity	0.61	
Adaptation measures can effectively prevent negative effects induced by water scarcity	0.87	
**Ascription of responsibility (α = 0.741, CR = 0.856, AVE = 0.664)**		
Every member of society should accept responsibility for adapting to water scarcity	0.77	^48^
The government (rather than farmers) should take more actions to adapt to water scarcity (reverse item)	0.85	
Other villagers, particularly big farmers (rather than me), should adapt to water scarcity (reverse item)	0.52	
**How do you feel about the following emotion when you decided to adapt to water scarcity?**		
Pride (α = 0.951, CR = 0.968, AVE = 0.91)		
I feel satisfied about not adapting to water scarcity	0.90	^42^
I feel self- worth when not adapting to water scarcity	0.95	
I feel pride when not adapting to water scarcity	0.94	
**How do you feel about the following emotion when you don’t decide to adapt water scarcity?**		
Guilt (α = 0.733, CR = 0.846, AVE = 0.647)		
I feel sadness when not adapting to water scarcity	0.61	^49^
I feel guilty when not adapting to water scarcity	0.74	
I feel shame when not adapting to water scarcity	0.73	
**Attitude (α = 0.931, CR = 0.95, AVE = 0.827)**		
Being involved in adapting water scarcity on my farm will be extremely valuable	0.86	^28,50^
Being involved in adapting to water scarcity on my farm is very necessary	0.86	
Being involved in adaptation to water scarcity in my farm is highly useful	0.85	
Being involved in adapting to water scarcity in my farm is completely rational	0.90	
**Subjective norm (α = 0.853, CR = 0.931, AVE = 0.871)**		
Society expects me to use less water on my farm	0.82	^28,51^
Most people who are important to me think I should adapt to water scarcity on my farm	0.91	
**Personal norms (α = 0.716, CR = 0.841, AVE = 0.639)**		
I feel personally obligated to do whatever I can to respond to water scarcity	0.62	^40,48,49,52^
I feel morally obliged to adapt to water scarcity, regardless of what others do	0.63	
I feel I carried out my obligation to deal with water scarcity if I use less water in my farm	0.78	
**Adaptation intention (α = 0.914, CR = 0.946, AVE = 0.853)**		
I will try to adapt to water scarcity in the next month	0.86	^28,50^
I plan to adapt with water scarcity in the next month	0.91	
I intend to engage in adaptive behavior in the next month	0.87	

Response scale (1–5); Strongly disagree–Strongly agree.

α = Cronbach's Alpha, CR = Composite Reliability, AVE = Average Variance Extracted.

### Data analysis

The structural equation modeling (SEM) technique was used to test the research models. SEM is a multivariate statistical analysis method used to analyze relationships between constructs with multiple items. Two basic statistical methods were used for testing SEM: covariance-based SEM and variance-based partial least square (PLS). Covariance-based modeling is suitable for model validation and comparison, while PLS is used for complex structural models with a large number of constructs^53^, hence the use of the covariance-based SEM in the study. The study also applied Amos 21.0 version software as a tool for conducting covariance-based SEM. The two-step approach of Anderson and Gerbing^54^ was used to carry out the SEM. The first step was to perform a confirmatory factor analysis (CFA) to obtain a satisfactory measurement model, and the second was to develop a structural model and test it.

### Verification of measurement and structural models

To evaluate the construct validity of measurement models, convergent and discriminant validity were used. Convergent validity means that two or more items related to a construct are theoretically related to each other. Three indices of factor loading, average variance extracted (AVE), and composite reliability (CR) were used to assess convergent validity. To confirm convergent validity, the values of the factor loading for each item and the AVE values for each latent construct should be greater than the threshold of 0.5. Additionally, convergent validity was confirmed with CR in our study as being above the acceptable threshold of 0.7^47^.

Furthermore, discriminant validity means that two or more constructs should not theoretically be related to each other. Discriminant validity is confirmed when the square root of AVE values for two latent constructs is greater than the correlation between the two constructs^55^.

Relative chi-square (chi-square/df) of less than 5 indicates good fit, relative chi-square of less than 3 indicates a good fit^56^. A root mean square error of approximation (RMSEA) below 0.08 indicates a goodness of fit, and finally a CFI, IFI, and NFI with a minimum value of 0.9 indicate an acceptable model^57^.

### Ethical approval

All procedures performed in studies involving human participants were in accordance with the ethical standards of the institutional and/or national research committee and with the 1964 Helsinki declaration and its later amendments or comparable ethical standards. 

### Informed consent

Informed consent was obtained from all subjects involved in the study. All materials and methods are performed in accordance with the instructions and regulations and this research has been approved by a committee at University of Zanjan, Iran.

## Results

### Respondents’ characteristics

The mean age of the farmers was 44.8 years (SD = 11.98). The youngest farmer was 19 and the oldest 75. The average number of farmers' family members was about 6 (SD = 2.94). The mean of agricultural experience was about 20 years (SD = 12.03). According to the survey, the minimum amount of farmers' land was 0.5 hectares and the maximum 200 hectares. Each farmer had an average of 15.16 hectares of land (SD = 20.59). 85.8% of farmers were married and 14.2% were single. 24.6% of the farmers were educated to diploma level. The frequency distribution of type of land ownership shows that 16% (39) of the respondents rented their land, 79.5% (194) owned the agricultural land they cultivated, and 4.5% of the respondents both rented and owned land. While, the majority of the participants (24.6%) had a diploma degree, 25 farmers (11.8%) had high school level education, 33 farmers (15.6%) elementary level education, 17 farmers (8.1%) middle school level education, and 59 farmers (28%) had a college degree.

### Measurement model analysis

This study tested three measurement models for each of models TRA, NAM, and the integrated model. As shown in Table 1, the factor loading of the items are in the 0.61–0.95 range, that is, within the acceptable range. The AVE values of each construct are also in the 0.639–0.91 range, which is higher than the recommended threshold. The minimum CR value was 0.841 which exceeded the recommended threshold of 0.7. As shown in Table 2, the square root AVE of each construct (bolded elements) are higher than the correlation of that construct with other constructs. Therefore, the research measurement model has acceptable discriminant validity. The fit indices of the measurement model of the three research models are shown in Table 3.

**Table 2:** Correlation between constructs and discriminant validity.

	AC	AR	P	G	AT	SN	PN	IN
Awareness of consequences	**0.825**							
Aspiration responsibility	0.012	**0.815**						
Pride	− 0.039	0.091	**0.954**					
Guilt	0.088	0.152	− 0.498	**0.805**				
Attitude	0.03	0.282	0.083	0.126	**0.91**			
Subjective norm	0.165	0.12	0.35	− 0.387	0.283	**0.933**		
Personal norm	0.172	0.28	0.321	− 0.183	0.363	0.501	**0.799**	
Intention	0.24	0.183	0.161	− 0.075	0.508	0.458	0.5	**0.923**

The square root AVE of each latent variable is bolded.

**Table 3:** Fit index.

Indexes	NAM		TRA		Integrated NAM and TRA		
	Measurement model	Structural model	Measurement model	Structural model	Measurement model	Structural model	Recommended value
CMIN	1.467	1.471	2.145	2.145	1.738	1.738	< 5
RMSEA	0.043	0.044	0.068	0.068	0.054	0.054	< 0.10
AGFI	0.888	0.888	0.919	0.919	0.841	0.841	> 0.9
GFI	0.921	0.919	0.964	0.964	0.883	0.882	> 0.9
CFI	0.975	0.974	0.987	0.987	0.955	0.954	> 0.9
IFI	0.976	0.975	0.987	0.987	0.956	0.955	> 0.9
NFI	0.927	0.925	0.976	0.976	0.902	0.900	> 0.9

### Structural equation modeling

**Figure 2:**
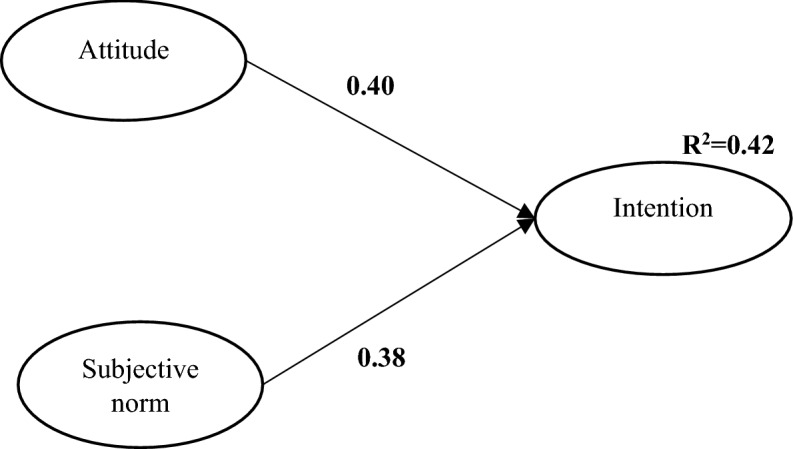
TRA model.

**Figure 3:**
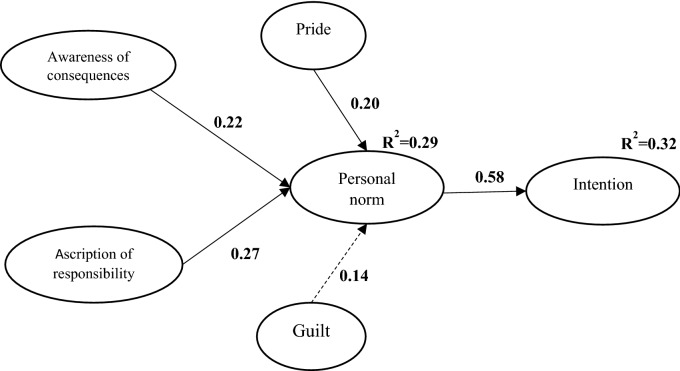
NAM model.

**Figure 4:**
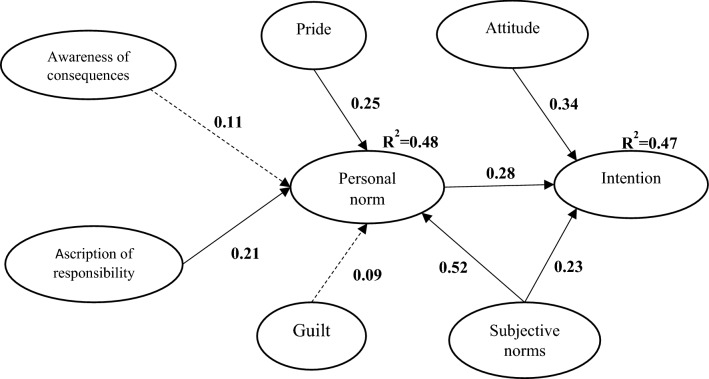
Integrated model.

Based on the results (Table 3), the structural models of the research have a good fit. The standardized path coefficients of the structural models TRA, NAM, and the integrated model are shown in Figs. 2, 3, and 4. In the TRA model (Fig. 2), two constructs of attitude and subjective norm positively predict the intention toward adaptive behavior. The most important predictor of intention was attitude, with a path coefficient of 0.40 (*P* < 0.001), followed by subjective norm with path coefficient of 0.38 (*P* < 0.01). The TRA model was able to predict 42% of the variance changes in the farmers’ intention to implement adaptive behaviors.

In the NAM model, the three constructs of AC, AR, and sense of pride were able to predict 29% of the variance of PN (Fig. 3). The most important predictor of PN was AR with a path coefficient of 0.27 (*P* < 0.001), followed by AC and pride with path coefficients of 0.22 and 0.20, respectively.

In the integrated NAM/TRA model, subjective norm with a path coefficient of 0.52 was the strongest predictor of PN. Pride and AR with path coefficient of 0.25 and 0.21 also affected PN. These factors predicted 48% of the variance changes in the personal norm, which is a significant increase over the NAM model. PN with a path coefficient of 0.28, subjective norm with a path coefficient of 0.23, and attitude with a path coefficient of 0.34 were also able to influence the farmers’ intention to carry out adaptive behaviors. Hence, attitude was the most important predictor of farmers intention to adaptation (Table 4). The integrated research model predicts 47% of the variance of intention toward adaptation (Fig. 4).

**Table 4:** Results structural equation modeling.

Hypothesis (Integrated model)			Unstandardized Regression Weights	SE	Standardized Regression Weights	C.R	*p* value	95% confidence interval	Results
AC→ PN			.087	.056	.105	1.541	.123	− .035− .252	*Reject*
AR→ PN			.105	.040	.208	2.629	.009	.022−.207	Support
P→ PN			.107	.039	.252	2.764	.006	.014– .232	Support
G→ PN			.065	.081	.093	.802	.422	− .11−.284	*Reject*
SN→ PN			.305	.057	.520	5.333	***	.144– .493	Support

## Discussion

This study used the two theories, NAM and TRA, singly and in integrated form, to better understand farmers’ intention to perform adaptation behavior in Khuzestan province. SEM analysis revealed that NAM, TRA, and the combined model can predict 32%, 42%, and 47%, respectively, of changes in farmers' intention toward adaptation. From a theoretical standpoint, the amount of intention prediction is acceptable in all three models. Armitage and Conner^58^ in their meta-analyses discussed the ability of psychological models to predict behavior. They found that TPB explained on average 39% and 27% of the variance in intention and behaviors, respectively. In other words, if we consider the findings of the meta-analysis of Armitage and Conner^58^ as a threshold, all three models used in this research have predicted the level of farmers' intentions at an appropriate level.

In earlier studies, Bamberg and Schmidt^25^ found that the theory of planned behavior (TPB model), a successor of the TRA, to be considerably more suitable than NAM. In the TRA, both attitude and subjective norm positively determine intention. In the NAM model, AC, AR and pride positively determine PN, guilt does not have a significant effect on PN, and, in turn, PN directly influences intention. In the combined model, AR, pride and subjective norm positively determine PN, while AC and guilt do not have a significant effect on PN and, in turn, attitude, subjective norm, and PN directly influence intention. In the combined model, the effect of PN was larger than that of subjective norm. This is in line with the finding of Niemiec et al.^23^ who, in their meta-analysis found that when both PN and subjective norm were included in the model, the PN had larger significant effect on intention. Daniel et al.^20^ also found that attitude towards water-related technology or behaviour is the most important psychological factor to make people treat the drinking water. Savari et al^59^ showed that denial of responsibility has negative effect on PN. Our results also revealed that both subjective norm and PN have significant effect on intention, consistent with relevant literature (39, ^60^). Bamberg and Moser^38^ in their meta-analysis found that when both variables were included together in the model, subjective norm, unlike PN, did not have a significant effect on intention. We can therefore conclude that findings of Bamberg and Moser^38^ in all contexts or samples could not be true. The general conclusion that can be drawn from this part of the study is that, from the perspective of Iranian farmers, adaptation is more of an economic activity to earn more profit on the farm than a moral act. However, after economic gain, adaptation is also a moral decision for them. Based on the view of Lindenberg and Steg^61^ of people's goals in performing environmental behaviors (hedonistic, gain, and normative goals), different goals might affect adaptation behavior among Iranian farmers. For them, the motivation to gain may be more salient, while normative goals may play a complementary role in adaptation behavior. Based on this we can conclude, like Bamberg and Moser^38^, that instead of being alternatives, these two models can be used as complementary ways of investigating pro-environmental behavior. The combination of profit and norm aspects in risk communication programs thus has a greater potential for those Iranian farmers wishing to implement adaptation activities (see also, Bamberg et al^32^).

Policy-wise, our findings show that the profit motive is the main factor in farmers' decisions to perform adaptive behavior. We thus recommend that risk communication messages and training programs provided by extension and advisory services should emphasize the fact that performing adaptive behavior reduces losses and increases profits. Describing and accentuating issues such as the benefits of adaptation, and the negative effects of non-adaptation, as well as increasing farmers' risk perceptions regarding the threats of climate change can be very effective in improving farmers' attitudes about adaptation. Similarly, as farmers are under social pressure to adapt, improving their attitudes, knowledge, and the risk perceptions of important pressure groups such as family, neighbors, peers, and colleagues about climate change and adaptation behaviors can have a significant impact on farmers' willingness to adapt. While PN is also a determinant of intention, increasing PN regarding adaptation behavior will probably increase farmers’ intention toward implementing adaptation strategies. In such cases the messages to farmers regarding the protection of water resources from the effects of climate change should emphasize the preservation of these resources for future generations and other living entities than humans. As the Muslim Holy Book, the Qur'an, implicitly and objectively considers the protection of natural resources, including water resources, as one of the duties of every Muslim, introducing the related verses of the Qur'an into training of advisory services can increase farmers' moral norm.

## Conclusion and limitation

The SEM analysis revealed firstly that the suggested model offers a reliable and practical exploratory framework to predict intention to adaptive behavior. The integrated model of TRA and NAM on the whole, to be suitable for explaining adaptation behavior in the specific context of Iranian farmers with the index explaining 47% of the variance in the adaptation. The power of the combined model, however, was better than that of the other two models, followed by TRA and then NAM. In addition, personal norm, subjective norm and attitude can directly predict intention. The result revealed that attitude was the greatest predictor of intention.

Although this research can have both theoretical and practical contributions, it still has its limitations. First of all, the research is descriptive and does not examine the relationship between cause and effect. It is also carried out based on self-reported information of farmers. The sample on which the research is based is from Khuzestan province in the southwest of Iran. Larger samples from all over Iran are needed if the results are to be generalized to the whole of the country. This study investigated farmers’ intentions to adaptation pattern based on the integrated model rather than their actual behavior. Existing studies indicate that behavioral intention models are robust across different domains of behavior, although actual behavior is not always equivalent to the attitudes of individuals or even to behavioral intentions^62^. Therefore, future studies should investigate farmers’ behaviors to adaptation.

## Data Availability

Some or all data, models, or code that support the findings of this study are available from the corresponding author upon reasonable request.
